# Anticancer drug delivery to cancer cells using alkyl amine-functionalized nanodiamond supraparticles†

**DOI:** 10.1039/c9na00453j

**Published:** 2019-08-28

**Authors:** Yue Yu, Xi Yang, Ming Liu, Masahiro Nishikawa, Takahiro Tei, Eijiro Miyako

**Affiliations:** Department of Materials and Chemistry, Nanomaterials Research Institute (NMRI), National Institute of Advanced Industrial Science and Technology (AIST) Central 5, 1-1-1 Higashi Tsukuba Ibaraki 305-8565 Japan e-miyako@jaist.ac.jp; Corporate Research Center, R&D Headquarters, Daicel Corporation 1239, Shinzaike, Aboshi-ku Himeji Hyogo 671-1283 Japan; Advanced Materials Planning, R&D Headquarters, Daicel Corporation 2-19-1 Konan, Minato-ku Tokyo 108-8230 Japan

## Abstract

Nanocarriers have attracted increasing interest due to their potential applications in anticancer drug delivery. In particular, the ability of nanodiamonds (NDs) to spontaneously self-assemble into unique nano-structured architectures has been exploited in the development of nanocarriers. In this context, we synthesized functional supraparticles (SPs) by the self-assembly of alkyl amine-modified NDs for use in anticancer chemotherapy. The structural, physical, and physiological properties of these ND-SPs as well as their high biocompatibility were assessed using microscopic techniques and various characterization experiments. Finally, a model anticancer drug (CPT; camptothecin) was loaded into the ND-SPs to investigate their anticancer efficacy *in vitro* and *in vivo*. After incubation of CPT-loaded ND-SPs with cancer cells, a dramatic anticancer effect of ND-SPs was expressed, compared to CPT-loaded ordinary nanocarriers of polyethylene glycol-modified polymer micelles and conventional Intralipid® 20% emulsions containing CPT. Our results demonstrated that ND-SPs may serve as a nanomedicine with significant therapeutic potential.

## Abstract 2

Nanocarriers have attracted increasing interest due to their potential applications in anticancer drug delivery.

## Graphical abstract

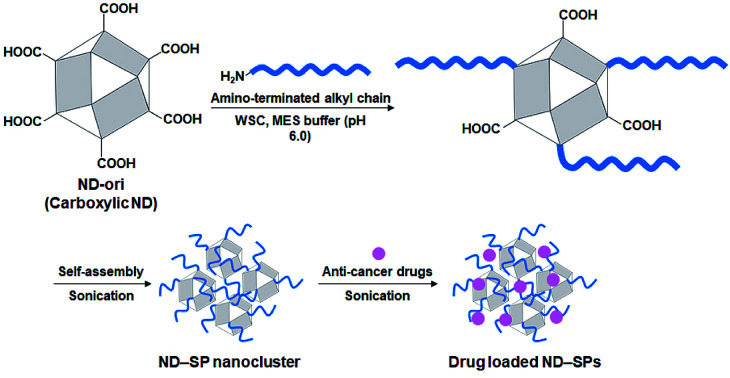

## Introduction

Various nanomaterials, such as liposomes, polymers, dendrimers, silicon, magnetic nanoparticles, and carbon materials, have been investigated as carriers in targeted anticancer drug delivery systems, using primarily the enhanced permeability and retention (EPR) effect, which is the preferential accumulation of nanoparticles within tumors owing to their leaky vasculature and poor lymphatic drainage.^1–3^ In particular, nanodiamonds (NDs), which have individual diameters of 2–10 nm and a truncated octahedral structure, have contributed significantly to the development of highly efficient and successful drug delivery systems due to their significant potential advantages, such as their relatively low cost, amenability to large-scale synthesis, unique optical properties, characteristic structures, and low toxicity.^4–7^

Self-assembled materials have gained great attention with respect to the design of drug carriers and three-dimensional architectures due to their wide range of applications from controlled drug delivery and tissue engineering to nanoelectronics.^8,9^ The ND surface possesses an assortment of functional groups, most of which are oxygenated and amino moieties, including carboxylic acid, lactone, ketone, ether, hydroxyl, and amino groups. These functional groups and/or the hydrophobic surface of NDs have mostly been used as a platform to conjugate with drug molecules through ionic, hydrogen, and hydrophobic interactions.^10–12^ However, the use of these functional groups as a starting point and a driving force for the self-organization of built up nanoconstructs is still not sufficiently exploited. We believe that these self-assembled formulations of NDs can be further expanded as a modification concept to deliver drugs to site-specific targets. To explore such possibilities, we recently demonstrated that perfluorooctanoic acid (PFOA)-chemically functionalized NDs spontaneously transformed into well-dispersed and biocompatible supraparticle (SP) nanoclusters that could serve as effective drug carriers for cancer treatment.^13^ PFOA was used as a model inducer to achieve the SP transformation *via* strong hydrophobic interactions. However, fluorine compounds, including PFOA, have potential environmental and health risks due to their accumulation and retention in biological bodies.^14,15^ Therefore, rational and new designs of ND-based SPs are desired for future clinical and biomedical applications.

In this context, we used a variety of alkyl amine derivatives, which have relatively lower biological retention, as alternative materials for PFOA to explore their capacity to form ND-SPs. Different lengths of alkyl chains of amine derivatives were modified onto a carboxylic acid-functionalized ND *via* simple covalent modification into self-assembled SPs that have well-controlled particle sizes. These SPs were further modified with an anticancer drug to investigate the potential improvement of the drug efficacy in comparison to conventional nanocarriers composed of polyethylene glycol (PEG)-modified polymer micelles and an ordinary drug carrier of Intralipid® 20% emulsions.

## Experimental

### ND-SP syntheses

NDs (diameter = 4–5 nm) were prepared *via* a detonation method.^16–18^ The synthesized NDs were purified with nitric acid and burned in a hydrogen gas atmosphere. Elemental analysis of the NDs (C (86.92%), H (0.44%), and N (2.29%) contents) was performed using an organic elemental analyzer (Micro Corder JM10, J-Science Lab Co., Ltd, Kyoto, Japan). The purified NDs were dispersed in distilled water *via* bead milling (Sand Grinder LSG-4U, Aimex Co., Ltd, Tokyo, Japan). The ND aqueous suspension was then centrifuged to remove water-insoluble NDs. The supernatant (ND-ori) was used for further studies. A 1 ml volume of ND-ori (ND concentration = 56 mg ml^−1^); 50 μl of *n*-octylamine (Oct), oleylamine (Ole), or 10 mg of dodecylamine (Dod) (FUJIFILM Wako Pure Chemical, Osaka, Japan); and 10 mg of 1-ethyl-3-(3-dimethylaminopropyl) carbodiimide hydrochloride (WSC) (FUJIFILM Wako Pure Chemical) were dissolved in 9 ml of 2-(*N*-morpholino)ethanesulfonic acid (MES) buffer (pH 6.0, 100 mM) *via* bath sonication (power output, 80 W; oscillation frequency, 40 kHz) (USD-2R, AS ONE, Osaka, Japan) for 5 min. After vigorous stirring for another 1.5 h at room temperature, the mixture was centrifuged (MX-307, TOMY, Tokyo, Japan) and washed three times with Milli-Q water to remove unreacted chemicals. The resultant pellet was resuspended in 10 ml of Milli-Q water *via* pulse-type sonication (VCX-600, Sonics, Danbury, CT, USA) for 10 min. The obtained ND-SP product was used for the subsequent experiments. The concentration of NDs (*ca.* 5.6 mg ml^−1^) in the final product was estimated using an ultraviolet (UV)-visible (Vis)-near infrared (NIR) spectrophotometer (V-730 BIO, Jasco, Tokyo, Japan). The amounts of Oct (*ca.* 13 wt%), Dod (*ca.* 17 wt%), and Ole (*ca.* 35 wt%) in the ND-SPs were analyzed *via* thermogravimetric analysis (TGA) (Q 500, TA Instruments, New Castle, DE, USA).

Camptothecin (CPT)-loaded Oct-ND-SP and Dod-ND-SP [CPT@Oct-ND-SP and CPT@Dod-ND-SP] complexes were prepared as follows. The washed Oct-ND-SP or Dod-ND-SP pellet was simply combined with 10 mg of CPT (FUJIFILM Wako Pure Chemical) followed by pulse-sonication in 10 ml of Milli-Q water for 10 min. CPT@ND-ori was prepared by mixing 10 mg of CPT, 1 ml of ND-ori, and 9 ml of Milli-Q water using a pulse-type sonicator for 10 min. CPT@PEGMEM, CPT@F127, CPT@DSPE-PEG, and CPT@Intralipid® 20% were obtained in the same manner except that the ND pellet was replaced with 56 mg of poly(ethylene glycol) methyl ether methacrylate (PEGMEM) (Sigma-Aldrich, St. Louis, MO, USA), 56 mg of pluronic F127 (F127) (FUJIFILM Wako Pure Chemical), 56 mg of *N*-(aminopropyl polyethyleneglycol)carbamyl-distearoylphosphatidyl-ethanolamine (DSPE-PEG) (Sunbright DSPE-020PA, Yuka Sangyo, Tokyo, Japan), or 1 ml of Intralipid® 20% (Sigma-Aldrich).

Boron dipyrromethene (BODIPY)-loaded Dod-ND-SP, PEGMEM, F127, and DSPE-PEG complexes [BODIPY@Dod-ND-SP, BODIPY@PEGMEM, BODIPY@F127, and BODIPY@DSPE-PEG] were prepared in a similar manner to the preparation of the CPT-loaded nanocomplexes.

### Characterization of the ND-SPs

The structure and morphology of the prepared ND-SPs were visualized using a high-resolution transmission electron microscope (TEM) (EM-002B, Topcon, Tokyo, Japan) at an accelerating voltage of 120 kV.

The hydrodynamic diameter of the ND-SPs was determined *via* dynamic light scattering (DLS) (Photal FPAR-1000, Otsuka Electronics, Osaka, Japan).

A UV-Vis-NIR spectrophotometer (V-730 BIO, Jasco, Tokyo, Japan) was used to measure the spectral profiles and concentrations of the ND-SPs and CPT complexes.

Fourier transform infrared (FTIR) spectroscopy (Spectrum One, PerkinElmer, Yokohama, Japan) analysis was carried out to identify the presence of alkyl chains on the ND surface.

### Cell culture and cytotoxicity evaluation

U2OS bone osteosarcoma and TIG-3 and MRC5 normal human fibroblast cell lines were obtained from the Japanese Collection of Research Bioresources Cell Bank (Tokyo, Japan) and cultured in Dulbecco's Modified Eagle's Medium (Gibco, Grand Island, NY, USA) containing 10% fetal bovine serum (Gibco), 2 mM l-glutamine (Gibco), 1 mM sodium pyruvate (Gibco), gentamycin (Gibco), penicillin–streptomycin (100 IU ml^−1^) (Gibco), and Hank's balanced salt solution (Life Technologies, Carlsbad, CA, USA). Cells were maintained at 37 °C in a humidified chamber at 5% CO_2_.

Cell viability was assessed using a Cell Counting Kit (CCK)-8 (Dojindo Laboratories, Kumamoto, Japan) according to the manufacturer's instructions. Briefly, cells were seeded in a 96-well plate (5 × 10^3^ cells per well) and allowed to attach overnight. Then, they were exposed to drugs or nanocomplexes as indicated. After washing with a fresh medium, the cells were incubated with the CCK-8 solution. A microplate reader (Infinite M200 PRO, Tecan, Männedorf, Switzerland) was used to read the absorbance at 450 nm.

### *In vitro* releasing behavior of CPT from ND-SPs

The CPT@Dod-ND-SP powder (5.6 mg) was dispersed in 1 ml of PBS buffer solution (pH 7.4) at 37 °C and stirred at 100 rpm. At determined time intervals, 50 μl of the solution was collected and mixed with 200 μl of dimethyl sulfoxide to completely dissolve the drugs. The mixture was then centrifuged at 15 000 rpm for 5 min to remove undissolved ND-SPs. The CPT concentration in the supernatant was finally analyzed using a UV-Vis-NIR spectrophotometer.

### Live cell imaging

U2OS cells were seeded in imaging dishes the night before treatment. The attached cells were incubated with BODIPY-loaded conventional nanocarriers (PEGMEM, F127, and DSPE-PEG) and BODIPY@Dod-ND-SPs for 3 h at 37 °C, followed by nuclear staining with Hoechst 33342 (1 μg ml^−1^; Thermo Fisher Scientific, Waltham, MA, USA) for 10 min. After three washes with PBS, the cells maintained in the RPMI 1640 phenol red-free medium (Thermo Fisher Scientific) were subjected to live-cell imaging. The images were acquired with a fluorescence microscope (IX73, Olympus, Tokyo, Japan) equipped with a mirror unit (IX3-FGFPXL, Olympus) and an electron-multiplying charge-coupled device camera (DP80, Olympus).

### *In vivo* antitumor experiments

All animal procedures were performed in accordance with the Guidelines for Care and Use of Laboratory Animals of National Institute of Advanced Industrial Science & Technology (AIST) and experiments were approved by the Animal Ethics Committee of AIST. Female BALB/cAJc-nu/nu mice (6 weeks old, 18 g) were bought from Japan SLC, Inc. (Shizuoka, Japan). Upon arrival, the mice were randomly separated into 3 groups (*n* = 4), and subcutaneously inoculated in the right flank with HT-29 cells (5 × 10^5^ cells per site) for the construction of the tumor xenograft model. Intraperitoneal injections of 200 μl of Dod-ND-SPs (56 mg kg^−1^), CPT@ND-SPs (CPT, 3 mg kg^−1^; ND-SPs, 56 mg kg^−1^) or PBS were performed every alternate day, when small tumor buds reach about 50 mm^3^. The overall health was monitored by body weight observation and the tumor size was calculated as *V* = *L* × *W*^2^/2, where *L* and *W* are the length and width of the tumor, respectively.

### Blood tests

The complete blood count and biochemical parameters were analyzed by Japan SLC, Inc. (Shizuoka, Japan) and Oriental Yeast Co., ltd. (Tokyo, Japan). Briefly, 10 week-old female BALB/cSlc mice (*n* = 5; average weight = 21 g; Japan SLC) were injected with 200 μl of sterilized water containing ND-SPs (ND-SP = 1.12 mg kg^−1^) or 200 μl of phosphate buffered saline (PBS) buffer *via* the tail vein. Blood samples were collected from the inferior vena cava of the mice after 4 weeks.

### Statistical analysis

The results are presented as the mean ± standard deviation of at least three independent experiments, with “*n*” indicating the number of samples per group. Differences between the groups were evaluated using Student's *t*-test for two groups and two-way analysis of variance for multiple groups. *, **, and *** denote *p* value is less than 0.05, 0.01, and 0.001, respectively.

## Results and discussion

### Synthesis and characterization of the ND-SPs

**Fig. 1:**
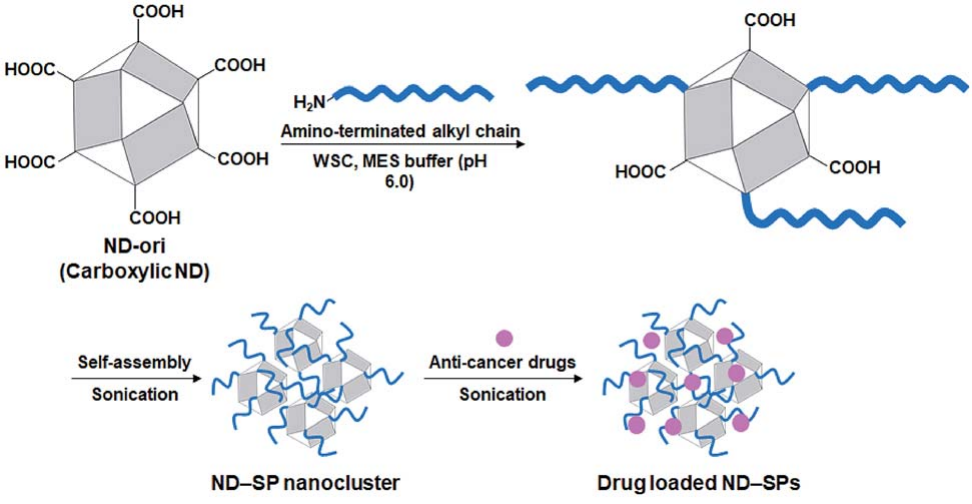
Schematic of ND-SP synthesis. Crude ND-ori was functionalized *via* cross-linking between surface carboxylic groups and the amino groups of alkyl chains *via* a condensation reaction followed by sonication to promote the self-assembly of ND-SP nanoclusters.

To align the effective nanocarrier features of the SPs with alkyl amine molecules, which have a relatively lower bioaccumulation compared to fluorine compounds, carboxylic ND (ND-ori)-based SPs (ND-SPs) were designed and synthesized *via* a condensation reaction between the carboxylic group on the surface of ND-ori and the amino group of the primary alkyl amines, *n*-octylamine (Oct), dodecylamine (Dod), and oleylamine (Ole), using WSC in an MES buffer (pH 6.0) (Fig. 1). The alkyl chains of the amines immediately trigger the formation of SPs due to their hydrophobic properties. Sonication allows the transformation of the self-assembled SP nanoclusters and the encapsulation of the anticancer drugs into the assembly. The simple preparation of ND-SPs, in addition to the convenient drug loading method, is beneficial for various biomedical applications.

**Fig. 2:**
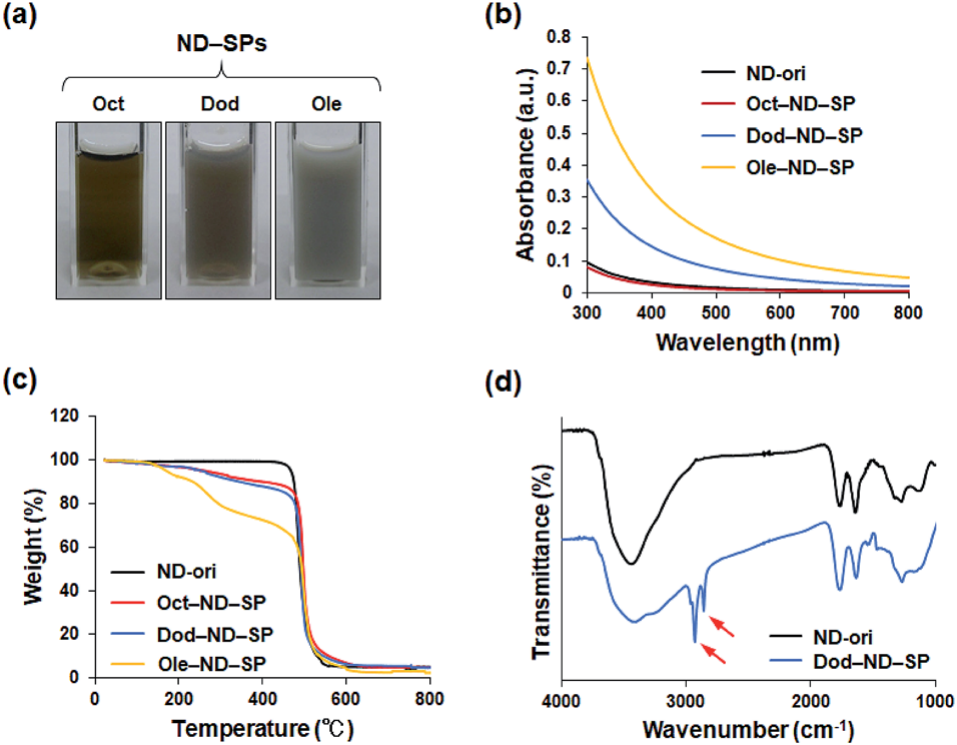
(a) Photos of ND-SP aqueous solutions. The concentration of the NDs is 5.6 mg ml^−1^. (b) UV-Vis-NIR absorbance spectra of ND-SPs. The concentration of the NDs is 56 μg ml^−1^. (c) TGA curves of ND-ori and various ND-SPs. (d) FTIR spectra of ND-ori and Dod-ND-SPs, identifying the Dod moiety in the conjugates. Red arrows represent the evidence of C–H stretch.

After the reaction, each of the ND-SP solutions, which have different alkyl chain lengths, showed unique colors and aspects (Fig. 2a). The turbidity of the samples became high due to surface scattering as the length of the alkyl chains increased.^19^ Indeed, the absorbances of the ND-SPs increased depending on their apparent turbidity (Fig. 2b). Of the solutions, the spectrum of Ole-ND-SPs showed the highest absorbance due to the suspension. Additionally, the TGA results revealed that approximately 13 wt%, 17 wt%, and 35 wt% of Oct, Dod, and Ole, respectively, were modified onto the ND surface (Fig. 2c). The FTIR peaks, indicated with arrows in Fig. 2D, can be clearly assigned to C–H stretch of the conjugated alkyl amines (Dod), further confirming the formation of ND-SPs.

**Fig. 3:**
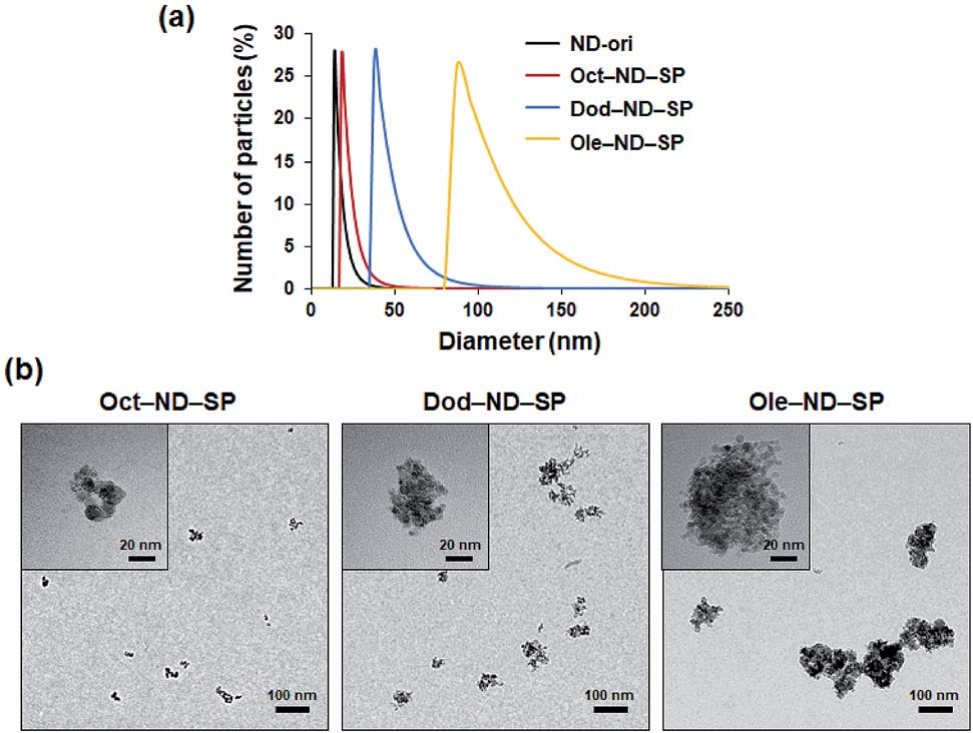
(a) DLS hydrodynamic size distribution of the ND-SPs. (b) TEM images of the ND-SPs. High magnification images are shown in the upper left corners of the panels.

Due to EPR effects, nano-scale (10–200 nm) particles are favorable for passively targeting tumors.^1–3^ Interestingly, according to the DLS measurements, the particle sizes of the prepared ND-SPs (*ca.* 18–87 nm) make them promising nanocarriers to express EPR effects (Fig. 3a). More interestingly, the diameters of ND-SPs can be easily controlled by changing the length of the alkyl chains. In fact, the DLS profiles of Oct-ND-SPs, Dod-ND-SPs, and Ole-ND-SPs displayed well-defined size distributions with different average diameters of 18 nm, 41 nm, 87 nm, respectively (Fig. 3a). The solutions remained stable for at least one week with no obvious change in size. The hydrodynamic diameters of the ND-SPs were higher than those of ND-ori (*ca.* 15 nm) due to the self-assembled nanocluster formations. The TEM observations clearly showed that the synthesized ND-SPs had cluster nanostructures based on the building blocks of ND-ori (Fig. 3b). The size of each ND-SP, estimated *via* TEM observations, depicted the same order of particle size as the DLS measurements. These results clearly indicate that alkyl amines can induce self-assembled SP formation of NDs *via* simple chemical functionalization.

**Fig. 4:**
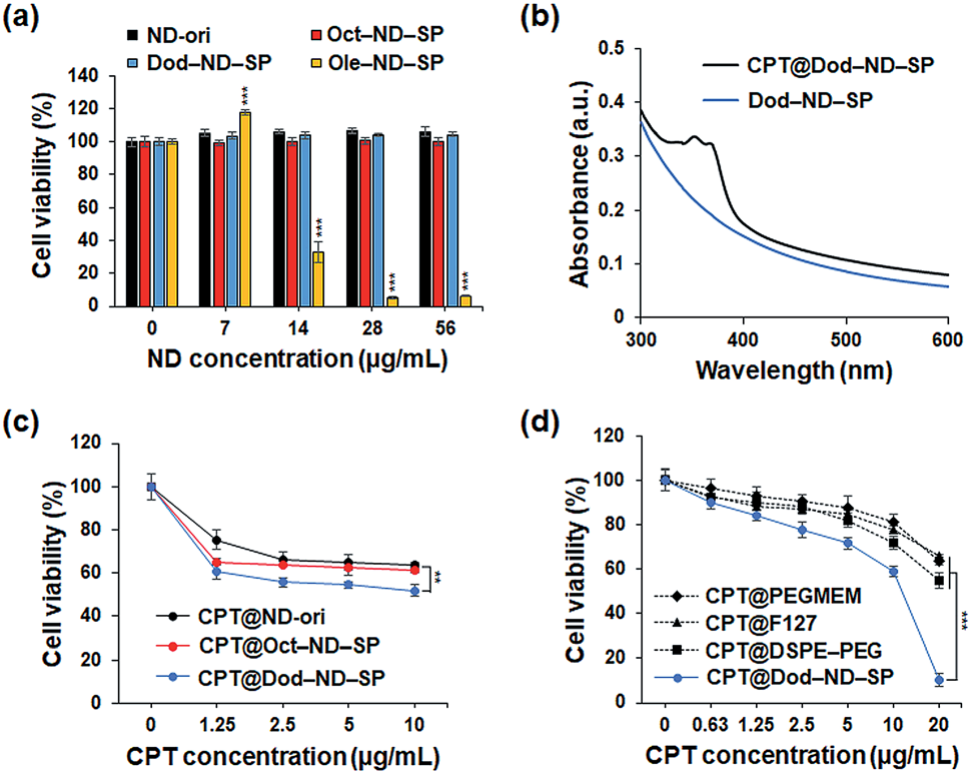
(a) Cytotoxicity evaluation of various ND-SPs. The U2OS cell viability was assessed after 24 h of treatment. The data are presented as the mean ± SD (*n* = 5), ***p < 0.001 (Student's *t*-test to 0 μg ml^−1^). (b) UV-Vis-NIR absorbance spectra of ND-SPs before (blue curve) and after (black curve) CPT incorporation. (c) Cell inhibitory effect of CPT@ND-ori, CPT@Oct-ND-SPs, and CPT@Dod-ND-SPs. The U2OS cancer cells were treated with the nanocomplexes for 24 h. The data are presented as the mean ± SD (*n* = 5), **p < 0.01 (two-way ANOVA test). (d) Cytotoxicity evaluation of the CPT-loaded conventional nanocarriers (PEGMEM, F127, and DSPE-PEG) and CPT@Dod-ND-SPs in the U2OS cells after 24 h of treatment. The data are presented as the mean ± SD (*n* = 6), ***p < 0.001 (two-way ANOVA test).

Next, the cytotoxicity of the ND-SPs was investigated because this is a very important issue for future clinical and biomedical applications. CCK-8 assays were used to analyze the survival of the U2OS cells (Fig. 4a). The U2OS cells were pre-incubated with five different ND concentrations (ND = 0 μg ml^−1^, 7 μg ml^−1^, 14 μg ml^−1^, 28 μg ml^−1^, and 56 μg ml^−1^) using the three types of ND-SPs. Over 98% of the cells were viable following the treatment with Oct-ND-SPs or Dod-ND-SPs at all concentrations. Additionally, Ole-ND-SPs displayed a higher cytotoxicity of more than 14 μg ml^−1^ of ND, likely due to the excess and stronger interaction between the cell surface and the longer alkyl chains of Ole, resulting in the denaturation of the cell membrane. Accordingly, Oct-ND-SPs and Dod-ND-SPs were used for further drug efficacy tests due to their low cytotoxicity.

**Fig. 5:**
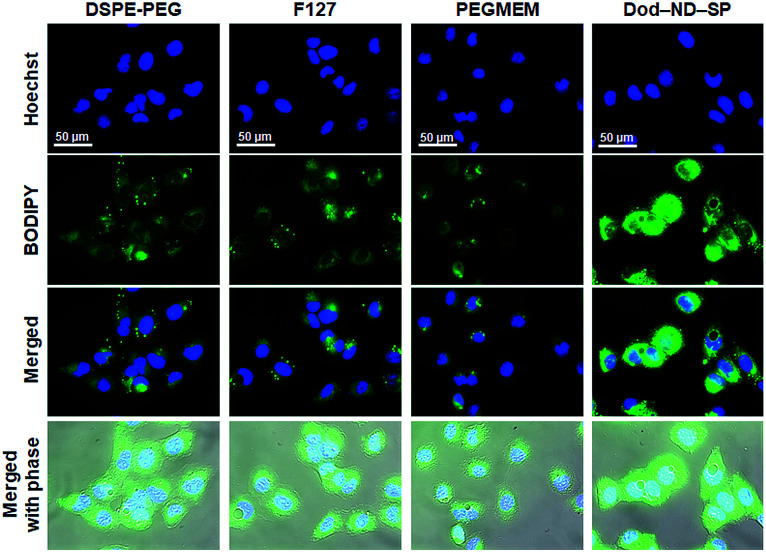
Fluorescence micrographs of U2OS cells incubated with BODIPY@DSPE-PEG, BODIPY@F127, BODIPY@PEGMEM, and BODIPY@Dod-ND-SPs for 3 h. Concentrations of BODIPY and nanomaterials are 10 μg ml^−1^ and 56 μg ml^−1^, respectively.

The drug CPT has a wide range of antitumor effects on cancers.^20^ CPT-based drugs are specific inhibitors of topoisomerase 1, leading to the destruction of DNA, and are currently being used as useful chemotherapeutic agents in clinical antitumor treatments. The CPT molecules were encapsulated in the ND-SPs *via* simple sonication, as shown in Fig. 1. The UV-Vis-NIR spectra displayed characteristic peaks of CPT at 350 nm and 369 nm after encapsulation (Fig. 4b). To test the anticancer drug efficacy of the functionalized ND-SPs, we incubated the U2OS cells with CPT-loaded ND-SPs [CPT@Oct-ND-SPs or CPT@Dod-ND-SPs] or CPT-loaded ND-ori (CPT@ND-ori) for 24 h. After washing with a fresh growth medium, the cell viability was analyzed using a CCK-8 kit. CPT@Dod-ND-SPs showed the highest anticancer chemotherapeutic effect (*ca.* 49%) at 10 μg ml^−1^ of CPT compared to the maximum drug efficacy values of the other materials [CPT@Oct-ND-SPs at ∼39% and CPT@ND-ori at ∼36%] (Fig. 4c). Interestingly, CPT@Dod-ND-SPs did not exhibit strong drug efficacy on TIG-3 and MRC5 normal fibroblast cell lines in comparison with U2OS osteosarcoma cells because of its potential targeting effect (Fig. S1a†). Besides, Dod-ND-SPs themselves do not have any cytotoxicity against these fibroblast cell lines (Fig. S1b†). More surprisingly, the most dramatic decrease in cell viability (down to 10%) was caused by increasing the CPT concentration to 20 μg ml^−1^, where CPT@DOD-ND-SPs exhibited better anticancer efficacy than conventional nanocarriers such as PEGMEM-, F127-, and DSPE-PEG-based polymer micelles including CPT molecules. It is well known that carbon-based nanomaterials have high biological affinities against cells.^21–23^ Additionally, the lower drug efficacies of conventional nanocarriers are likely due to PEG moieties on their constructs that generally inhibit interactions with cells.^24,25^ To clarify the effective transmembrane permeation properties of ND-SPs, fluorescence live cell imaging was performed (Fig. 5). A hydrophobic fluorescent molecule (BODIPY) was co-assembled with ND-SPs *via* non-covalent hydrophobic interaction. Fluorescence microscopy showed that compared to PEGMEM-, F127-, and DSPE-PEG-based polymer micelles, Dod-ND-SPs were more efficiently internalized by the U2OS cells at 37 °C. Furthermore, the efficacy of CPT suspended in Intralipid® 20% emulsion, which is one of the recommended drug carriers, was also compared with the anticancer effect of CPT@Dod-ND-SPs (Fig. S2†). Dod-ND-SPs showed a stronger drug efficacy than Intralipid® 20%. These results clearly indicate that drug-loaded functional ND-SPs can effectively eliminate cancer cells.

The final goal of this research is to build a functional drug delivery system of ND-SPs. Herein, we have calculated the loading capacity and efficiency (Fig. S3a†). *In vitro* release profiles of CPT molecules from Dod-ND-SPs were monitored in PBS buffer (pH 7.4) (Fig. S3b†). CPT molecules were rapidly released from Dod-ND-SPs over time, where it reached a plateau after about 24 h, and were released up to 75% in PBS after 72 h.

**Fig. 6:**
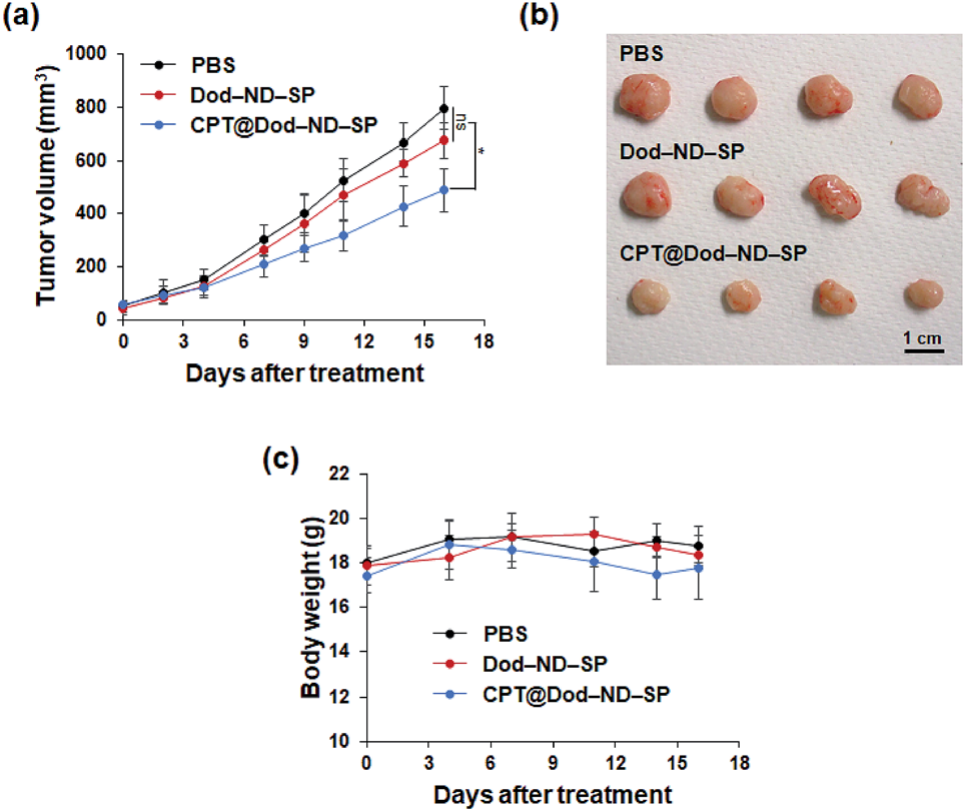
*In vivo* anticancer effects. (a) Tumor growth curves of mice after intraperitoneal injection of CPT@Dod-ND-SP (CPT, 3 mg kg^−1^; ND-SP, 56 mg kg−1); Dod-ND-SPs (56 mg kg^−1^), and PBS (vehicle control). The data are presented as the mean ± SD (*n* = 4), *p < 0.05 (two-way ANOVA test). (b) Images of tumors isolated from mice at the end of the experiment. (c) Average body weight of different groups of mice during treatment.

To demonstrate the feasibility of CPT-loaded ND-SPs for cancer treatment *in vivo*, the antitumor activity of CPT@Dod-ND-SPs was evaluated using HT-29 tumor xenograft models. As shown in Fig. 6A, the tumor growth was strongly suppressed in the CPT@Dod-ND-SP treatment group as compared to the PBS control, although Dod-ND-SPs alone didn't show significant difference. At the end of treatment, the tumor size of the mice receiving CPT@Dod-ND-SPs was remarkably smaller than those receiving PBS (Fig. 6B), suggesting that CPT@Dod-ND-SPs can retard tumor progression effectively. In addition, there was no significant loss of body weight in the mice (Fig. 6C), demonstrating that the systemic toxicity of CPT@Dod-ND-SPs is negligible.

To investigate the biocompatibility of ND-SPs further, mice were intravenously administered with sterilized water containing Dod-ND-SPs (200 μl; Dod-ND-SPs, 1.12 mg kg^−1^) or 200 μl of the PBS buffer for 4 weeks and subsequent blood tests were performed (Tables 1 and 2). The hematological and biochemical parameters did not differ between the mice intravenously injected with Dod-ND-SPs and PBS, confirming the absence of an inflammatory response or systemic side effects and underscoring the biocompatibility of ND-SPs.

**Table 1:** Hematological tests of mice injected with PBS or Dod-ND-SPs after 4 weeks^a^

Entry	Unit	PBS (*n* = 5)	Dod-ND-SPs (*n* = 5)	*p* value
WBC	×10^2^ μl^−1^	85.3 ± 13.4	92.0 ± 16.7	>0.05
RBC	×10^4^ μl^−1^	923.8 ± 21.9	938.4 ± 49.7	>0.05
HGB	g dl^−1^	13.9 ± 0.6	14.3 ± 0.8	>0.05
HCT	%	41.4 ± 1.9	43.0 ± 2.5	>0.05
MCV	fl	45.7 ± 0.5	45.9 ± 0.5	>0.05
MCH	pg	15.3 ± 0.2	15.2 ± 0.1	>0.05
MCHC	g dl^−1^	33.5 ± 0.6	33.2 ± 0.5	>0.05
PLT	×10^4^ μl^−1^	64.5 ± 11.0	70.1 ± 2.7	>0.05

a Results represent the mean ± standard deviation of five experiments. Statistical analyses were performed using Student's *t*-test. Abbreviations: WBC, white blood cells; RBC, red blood cells; HGB, hemoglobin; HCT, hematocrit; MCV, mean cell volume; MCH, mean cell hemoglobin; MCHC, mean cell hemoglobin concentration; and PLT, platelets.

**Table 2:** Biochemical tests of mice injected with PBS or Dod-ND-SPs after 4 weeks^a^

Entry	Unit	PBS (*n* = 5)	Dod-ND-SPs (*n* = 5)	*p* value *vs.* PBS
CRP	mg ml^−1^	1.2 ± 0.2	0.9 ± 0.2	>0.05
TP	g dl^−1^	3.9 ± 0.2	4.1 ± 0.1	>0.05
ALB	g dl^−1^	2.6 ± 0.2	2.8 ± 0.0	>0.05
BUN	mg dl^−1^	21.6 ± 0.6	23.5 ± 3.1	>0.05
CRE	mg dl^−1^	0.13 ± 0.02	0.12 ± 0.01	>0.05
Na	meq. l^−1^	145.6 ± 1.4	148.6 ± 0.5	>0.05
K	meq. l^−1^	3.9 ± 0.4	3.4 ± 0.2	>0.05
Cl	meq. l^−1^	113.6 ± 1.0	117.0 ± 0.9	>0.05
AST	IU l^−1^	59.4 ± 11.0	70.5 ± 11.0	>0.05
ALT	IU l^−1^	31.2 ± 5.0	39.0 ± 7.2	>0.05
LDH	IU l^−1^	230.0 ± 41.0	272.5 ± 44.8	>0.05
AMY	IU l^−1^	1597.0 ± 133.9	1624.2 ± 75.7	>0.05
CK	IU l^−1^	158.8 ± 45.4	166.4 ± 59.1	>0.05

a Results represent the mean ± standard deviation of five experiments. Statistical analyses were performed using Student's *t*-test. Abbreviations: CRP, C-reactive protein; TP, total protein; ALB, albumin; BUN, blood urea nitrogen; CRE, creatinine; AST, aspartate aminotransferase; ALT, alanine transferase; LDH, lactate dehydrogenase; AMY, amylase; and CK, creatine kinase.

## Conclusions

We demonstrated the spontaneous self-assembly of commercially available alkyl amine-modified NDs into unique geometrical architectures leading to the formation of SPs for the future treatment of osteosarcoma. We conducted a detailed investigation of SP formation for different lengths of alkyl chains of the primary amines. The structural, physical, and physiological properties of these ND-SPs were assessed using microscopic techniques and optical, thermal gravimetric, and cell tests. The sizes of the ND-SPs were easily controlled by changing the length of the alkyl chains of the amines. A model anticancer drug CPT was incorporated into these ND-SPs and conventional PEG-modified polymer micelles, and the ND-SPs showed good drug efficacy against U2OS bone osteosarcoma cells. In particular, CPT-loaded ND-SPs displayed the highest anticancer therapeutic effect. *In vivo* anti-tumor assays showed that CPT delivered by ND-SPs caused stronger tumor suppression of HT-29 colorectal adenocarcinoma xenografts. These self-assembled ND-SPs open up possibilities for various applications and will be explored further for drug delivery. Compared to ordinary nanocarriers, ND-based materials generally possess the overwhelming superiorities of easy size control, robustness against chemicals and physical conditions, good thermal stability, and unique optical properties.^26,27^ However, the investigation of ND-SPs as drug carriers is still in its infancy. In this regard, developing ND-SPs is highly promising for various clinical and biological applications for cancer therapy.

## Conflicts of interest

There are no conflicts to declare.

## Supplementary Material

NA-001-C9NA00453J-s001
