# Supplementary material for: Anticancer drug delivery to cancer cells using alkyl amine-functionalized nanodiamond supraparticles
Source: Nanoscale Adv. 2019 Aug 28;1(9):3406–12. doi: 10.1039/c9na00453j (PMC9417144; doi:10.1039/c9na00453j)
Supplement: NA-001-C9NA00453J-s001 [file NA-001-C9NA00453J-s001.pdf]

Electronic Supplementary Material (ESI) for Nanoscale Advances.
This journal is © The Royal Society of Chemistry 2019

Supporting Information for:

Anticancer drug delivery to cancer cells using alkyl amine-functionalized nanodiamond supraparticles

Yue Yu,[ab] Xi Yang,[ab] Masahiro Nishikawa,[c] Ming Liu,[c] Takahiro Tei,[d] and Eijiro Miyako[ab]*

a. Nanomaterials Research Institute (NMRI), National Institute of Advanced Industrial Science and Technology (AIST), Central 5, 1-1-1 Higashi, Tsukuba, Ibaraki 305-8565, Japan.

b. Present address: School of Materials Science, Japan Advanced Institute of Science and Technology, 1-1 Asahidai, Nomi, Ishikawa, 923-1292, Japan.

c. Corporate Research Center, R&D Headquarters, Daicel Corporation, 1239, Shinzaike, Aboshi-ku, Himeji, Hyogo 671-1283, Japan.

d. Advanced Materials Planning, R&D Headquarters, Daicel Corporation, 2-19-1 Konan, Minato-ku, Tokyo 108-8230, Japan.

* To whom correspondence should be addressed. E-mail: e-miyako@jaist.ac.jp

Supporting Figures

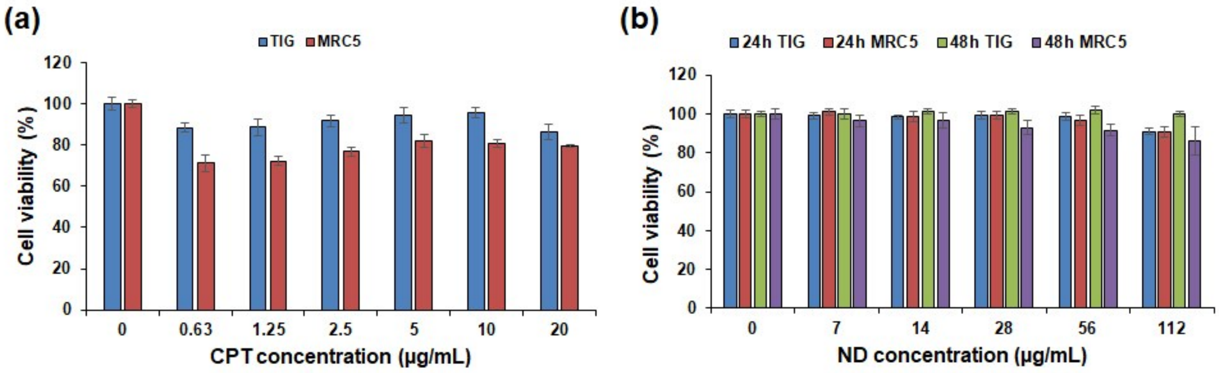

Figure S1. (a) Drug efficacy of CPT@Dod-ND-SPs on normal cell lines. The TIG-3 and MRC5 fibroblast cell viabilities were tested after 24 h of exposure with CPT@Dod-ND-SPs. Data represent the mean of five determinations; error bars show the SD. (b) Cytotoxicity test of Dod-ND-SPs on normal cell lines. The TIG-3 and MRC5 fibroblast cell viabilities were tested after 24 h and 48 h of treatment. Data represent the mean of five determinations; error bars show the SD.

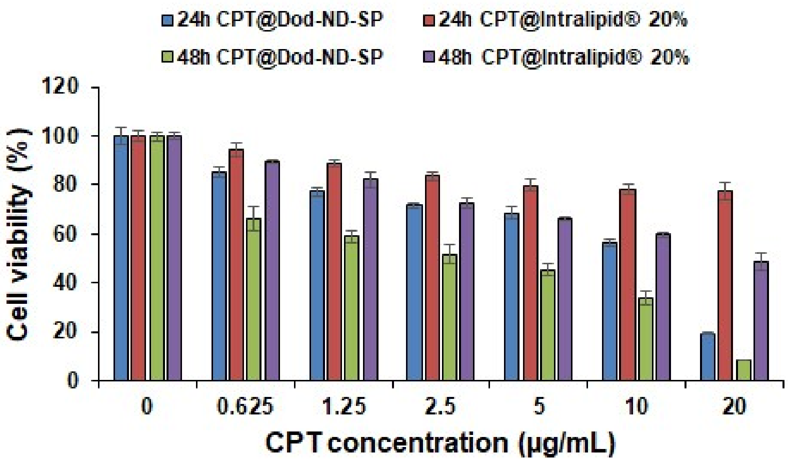

Figure S2. *In vitro* anticancer effect of ND-SPs and Intralipid® 20% as a drug delivery carrier. Cytotoxicity evaluation of ND-SPs and Intralipid® 20% at various CPT concentration. U2OS cell viability was tested after 24 h and 48 h of treatment.

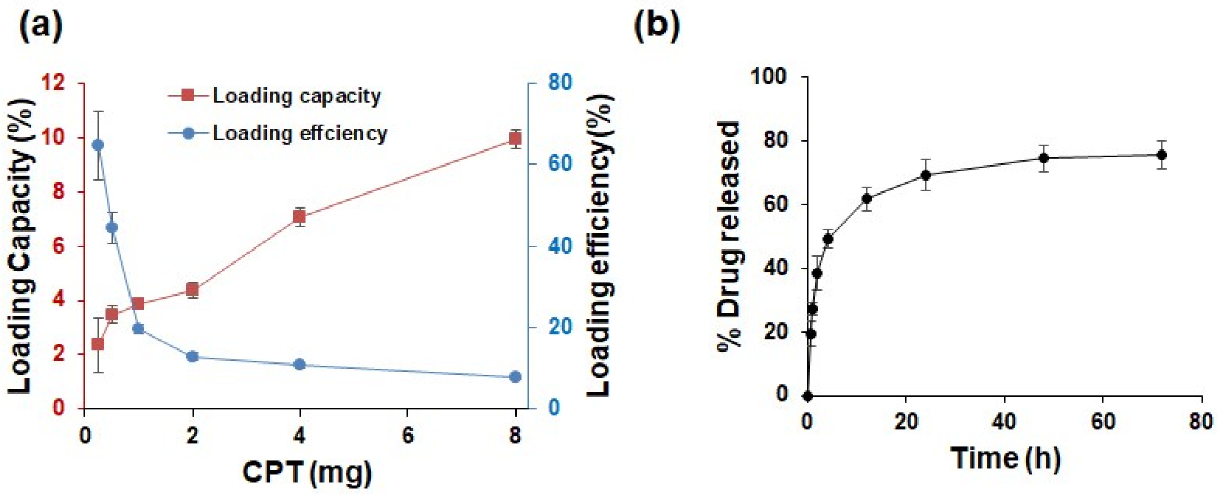

Figure S3. (a) Lading capacity and loading efficiency of CPT molecules into ND-SPs. (b) CPT releasing profile from Dod-ND-SPs.
